# *KRAS* and *GNAS* mutations in cell‐free DNA and in circulating epithelial cells in patients with intraductal papillary mucinous neoplasms—an observational pilot study

**DOI:** 10.1002/1878-0261.13719

**Published:** 2024-09-01

**Authors:** Christine Nitschke, Marie Tölle, Philipp Walter, Kira Meißner, Mara Goetz, Jolanthe Kropidlowski, Andreas W. Berger, Jakob R. Izbicki, Felix Nickel, Thilo Hackert, Klaus Pantel, Harriet Wikman, Faik G. Uzunoglu

**Affiliations:** ^1^ Department of General, Visceral and Thoracic Surgery University Hospital Hamburg‐Eppendorf Germany; ^2^ Mildred Scheel Cancer Career Center Hamburg Germany; ^3^ Institute of Tumor Biology University Hospital Hamburg‐Eppendorf Germany; ^4^ Department of Tumor Biology University Hospital Hamburg‐Eppendorf Germany; ^5^ Department of Internal Medicine I Ulm University Germany; ^6^ Department of Internal Medicine II Evangelisches Krankenhaus Königin Elisabeth Herzberge Berlin Germany

**Keywords:** CEC, ddPCR, GNAS, IPMN, KRAS, liquid biopsy

## Abstract

Intraductal papillary mucinous neoplasms (IPMNs) are potential precursor lesions of pancreatic cancer. We assessed the efficacy of screening for KRAS proto‐oncogene, GTPase (*KRAS*), and GNAS complex locus (*GNAS*) mutations in cell‐free DNA (cfDNA)—using digital droplet polymerase chain reaction (ddPCR) and circulating epithelial cell (CEC) detection—as biomarkers for risk stratification in IPMN patients. We prospectively collected plasma samples from 25 resected patients at risk of malignant progression, and 23 under clinical surveillance. Our findings revealed *KRAS* mutations in 10.4% and *GNA*S mutations in 18.8% of the overall cohort. Among resected IPMN patients, *KRAS* and *GNAS* mutation detection rates were 16.0% and 32.0%, respectively, whereas both rates were 4.0% in conservatively managed IPMN. *GNAS* mutations in cfDNA were significantly more prevalent in resected IPMN (*P* = 0.024) compared with IPMN under surveillance. No CECs were detected. The absence of *KRAS* and *GNAS* mutations could be a reliable marker for branch duct IPMN without worrisome features. The emergence of *GNAS* mutations could prompt enhanced imaging surveillance. Neither the presence of established worrisome features nor *GNAS* or *KRAS* mutations appear effective in identifying high‐grade dysplasia among IPMN patients.

## Graphical abstract

This study demonstrates that *KRAS* and *GNAS* mutations are more prevalent in patients with resected intraductal papillary mucinous neoplasms (IPMN) compared to those under clinical surveillance. *GNAS* mutations significantly differ between the two patient cohorts, indicating that their absence may serve as a potential biomarker to support conservative management strategies for IPMN patients.

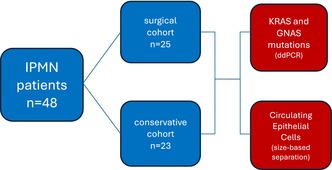

**Abbreviations**
AB: antibody
CEC: circulating epithelial cell
cfDNA: cell‐free DNA
CTC: circulating tumour cell
ctDNA: circulating tumour DNA
ddPCR: digital droplet polymerase chain reaction
IPMN: intraductal papillary mucinous neoplasm
MAF: mutant allele frequency
MRCP: magnetic resonance cholangiopancreatography
PDAC: pancreatic ductal adenocarcinoma
PFA: paraformaldehyde
spss
: Statistical Package for the Social Sciences

## 1 Introduction

Intraductal Papillary Mucinous Neoplasms (IPMN) of the pancreas are the most commonly encountered cystic lesions in clinical practice. They have been recognised as significant precursor lesions for invasive pancreatic ductal adenocarcinoma (PDAC). This connection underscores the critical need for precise risk assessment and timely intervention in managing IPMN patients to mitigate the progression to more severe pancreatic conditions effectively [1].

IPMN can be classified into three primary subtypes based on the involvement of the pancreatic duct system: main duct, branch duct and mixed type. Notably, the main duct and mixed type IPMN, which affect both the main and branch ducts, present the highest risk of progression to malignancy, with rates up to 70.0% [2]. Accurately distinguishing IPMN from other pancreatic cystic lesions and assessing their malignancy risk are critical steps in determining each patient's most appropriate management strategy. This strategy typically involves surgical resection for patients in the higher‐risk category or active surveillance for those with IPMN at a lower risk of malignant transformation.

Patients diagnosed with IPMN require close monitoring or surgical resection, depending on the IPMN subtype and other risk factors [3]. The medical imaging methods of choice for characterising the lesion and guiding the decision for surgical intervention are magnetic resonance cholangiopancreatography (MRCP) and endoscopic ultrasound. These techniques are instrumental in identifying IPMN subtypes and evaluating worrisome features, thereby determining which patients are most likely to benefit from surgical resection [4]. According to current guidelines, worrisome features are, for instance, the size of the cyst, the presence of solid components within the lesion, and the degree of dilatation of the pancreatic duct [3]. These features are particularly crucial in managing branch duct IPMN, as they correlate with an increased risk of malignant progression. However, reliance on imaging and definition of main duct/branch duct classification and worrisome features alone for decision‐making regarding surgical intervention may lead to inaccuracies and rushed decisions, resulting in a considerable number of unnecessary surgeries. This is a substantial concern, given the intrinsic risks and complications associated with pancreatic surgery [5, 6, 7].

There is a growing need for more precise and reliable diagnostic methods to address this issue to inform surgical decision‐making for IPMN. In recent years, liquid biopsy has emerged as a non‐invasive diagnostic method that has gained significant importance in cancer research [8]. This technique involves the analysis of disease‐related markers in various biofluids, such as blood or plasma, to obtain valuable information about the biology of the disease. Liquid biopsy holds great potential for contributing to personalised therapy by providing real‐time and minimally invasive monitoring of cancer progression [9].

The subject of many current studies is the detection of circulating tumour cells (CTCs) and circulating tumour DNA (ctDNA) through liquid biopsy in manifest PDAC [10]. One molecular alteration that plays a pivotal role in the progression of PDAC and its precursor lesions is the presence of *KRAS* mutation. *KRAS* is a G‐protein that regulates cell growth and differentiation. Mutations in the *KRAS* gene result in losing control over the protein, leading to unregulated cell proliferation, found in > 80.0% of PDAC patients [11]. Monitoring ctDNA with *KRAS* mutations has emerged as an independent prognostic marker with adverse implications for early and advanced PDAC cases [10]. In addition, mutations in the *GNAS* gene play a crucial role in IPMN development, affecting the cGMP‐mediated receptor signalling pathways [12].

Our study specifically targeted hotspot mutations present in *KRAS* (G12A, G12C, G12D, G12R, G12S, G12V and G13D) and *GNAS* (R201C) genes, which allow for highly sensitive and quantitative detection methods in IPMN patients [10, 13]. We utilised digital droplet polymerase chain reaction (ddPCR) to detect these specific mutations in cell‐free DNA (cfDNA) and concurrently screened for the presence of circulating epithelial cells (CECs) in patients either under surveillance or scheduled for surgery [14]. The primary objective was to evaluate the significance of cfDNA‐based analyses for assessing malignancy risk—particularly through the detection of *KRAS* and *GNAS* mutations—and to explore the role of CECs in enhancing clinical decision‐making strategies for IPMN treatment.

## 2 Materials and methods

### 2.1 Patient cohort

Our explorative study cohort prospectively included patients diagnosed with IPMN between November 2020 and June 2023 at the University Hospital Hamburg‐Eppendorf. During this period, *n* = 48 adult IPMN patients were enrolled in the study. Among them, *n* = 25 patients presented with main duct IPMN, mixed type or branch duct IPMN with worrisome features, and underwent surgery—representing the surgical cohort. The remaining *n* = 23 patients with branch duct IPMN and no worrisome features were closely conservatively monitored through our outpatient clinic—representing the conservative cohort. Clinicopathological data were collected from all patients. Additional data were provided from the prospectively surgical database for patients with pancreatic resections, which was in concordance with the General Data Protection Regulation guidelines. Clinical characteristics (e.g., age, gender, risk classification), preoperative diagnostics (e.g., imaging, tumour marker), operative details (e.g., type of resection performed), and follow‐up data were extracted from the database. The Ethics Commission of Hamburg approved collecting data and patient material for this study (PV3548). The study methodologies conformed to the standards set by the Declaration of Helsinki. The experiments were undertaken with the understanding and written consent of each subject.

### 2.2 Sample collection

Blood samples were collected from each patient in the study before initiating any treatment interventions. For each patient, peripheral blood samples were taken using four 7.5‐mL EDTA tubes to obtain plasma samples for the cfDNA analysis and for CEC detection.

### 2.3 cfDNA isolation from plasma

Plasma samples were isolated from whole blood through standard two‐step centrifugation (10 min 300 ***g*** and 10 min 1800 ***g***) and were stored at −80.0 °C. The isolation of cfDNA from plasma was performed using the QIAamp Circulating Nucleic Acid Kit (Qiagen, Hilden, Germany) following the corresponding protocol [10]. The concentration of cfDNA was quantified using the Qubit 4 Fluorometer (Thermo Fisher Scientific, Waltham, MA, USA) and was stored at −20.0 °C.

### 2.4 ddPCR for *KRAS* and *GNAS* mutations

DdPCR was employed to detect the hotspot mutations in the *KRAS* and *GNAS* genes. The ddPCR analysis was conducted using the Bio‐Rad ddPCR system and the manufacturer's protocol (Bio‐Rad Laboratories, Hercules, CA, USA) [15]. Specific primers (assays) and probes (qPCR Supermix) targeting the *KRAS* and *GNAS* hotspot mutations were utilised in the ddPCR reactions. For *KRAS*, the Bio‐Rad ‘*KRAS* G12/G13 Screening assay’ (*KRAS* mutations G12A, G12C, G12D, G12R, G12S, G12V, G13D) and for *GNAS* the Bio‐Rad assay ‘*GNAS* p.R201C’ were applied. Due to the limited amount of cfDNA in IPMN patients, our study focusses on the most prevalent *KRAS* mutations, such as G12D and G12V—and *GNAS* R201C [16, 17]. Each sample was tested in duplicates except for *n* = 10 samples due to insufficient cfDNA concentration (one cell tested). Each run included a nontemplate control, a wild‐type control, and a positive control (mutation known). To evaluate the positive mutant droplets, the PCR plates were read by the QX100 Droplet Reader using Quantasoft^®^ software version 1.7.4 (Bio‐Rad Laboratories, USA). For *KRAS*, a sample was considered positive if two or more droplets were positive (cut‐off 10 000); for *GNAS*, a sample was considered positive if two or more droplets were positive (cut‐off 6000). As described before, the absolute number of copies per mL of plasma was calculated [10].

### 2.5 CEC detection from EDTA blood

CECs in the blood samples for the first analysed *n* = 31 patients were detected through the marker‐independent microfluid‐based Parsortix™ cell separation system. Previous studies have shown that the Parsortix™ device provides size and deformability‐based enrichment by capturing cells sized > 6.5 μm [18]. Data on detecting CTCs and Cancer‐associated Macrophage‐like cells in blood samples from patients with PDAC through Parsortix™ have been recently reported [19].

All harvested cells were analysed via immunofluorescence staining for the nuclear staining DAPI, pan‐keratins as an epithelial marker for positive selection, and CD45 for negative enrichment. The enriched cell fraction was first fixed with 4.0% paraformaldehyde (PFA, Sigma, Ronkonkoma, NY, USA) for 10 min at room temperature for the immunofluorescence staining. Then, it was permeabilised with 0.2% Triton X‐100 (Sigma Aldrich, St. Louis, MO, USA) for 10 min, blocked with 10.0% AB‐Serum (Bio‐Rad, Contra Costa County, CA, USA), and incubated with DAPI (1 : 500), conjugated pan‐keratins C11 (1 : 80, AlexaFluor546 Cell Signalling, Danvers, MA, USA) and AE1/3 (1 : 80), Anti‐Pan‐CytokeratinAlexa Fluor 546 Clone (Invitrogen, Waltham, MA, USA), and allophycocyanin (APC) conjugated CD45 antibodies (1 : 150, Alexa Fluor 647 anti‐human CD45 Clone H130 BioLegend, SanDiego, MA, USA) for 60 min. The consecutive analysis was performed using immunofluorescence microscopy. A CEC was enumerated as positive using the definitions DAPI^+^, Keratin^+^, and CD45^−^.

After *n* = 31 patients, an interim analysis was performed, and due to negative findings, the CEC detection was terminated.

### 2.6 Statistical data analysis

The statistical analyses were performed using spss version 29 (SPSS Inc., Chicago, IL, USA). For the evaluation of a potential association between the *GNAS* and *KRAS* mutation status and clinicopathological parameters (including current risk classification), the chi‐squared/Fisher's exact test was used. Significant statements refer to *P*‐values of two‐tailed tests that were < 0.05. The sensitivity of mutations in detecting a surgical resected IPMN was determined by dividing the number of positive cases in the surgical cohort by the total number of cases in the surgical cohort. Similarly, the specificity in detecting low‐risk IPMNs in the surveillance cohort as wildtype mutations (negative) was calculated by dividing the number of negative cases in the surveillance cohort by the total number of cases in the surveillance cohort.

## 3 Results

The study cohort included 48 patients (25 resected IPMN patients and 23 conservatively managed IPMN patients). We analysed KRAS and GNAS mutations in their plasma samples using ddPCR (cfDNA) and quantified total cfDNA concentrations (ng·mL^−1^) in these plasma samples. We also examined the existence of CECs in the plasma samples of 31 IPMN patients.

### 3.1 *KRAS* and *GNAS* mutation status

**Fig. 1:**
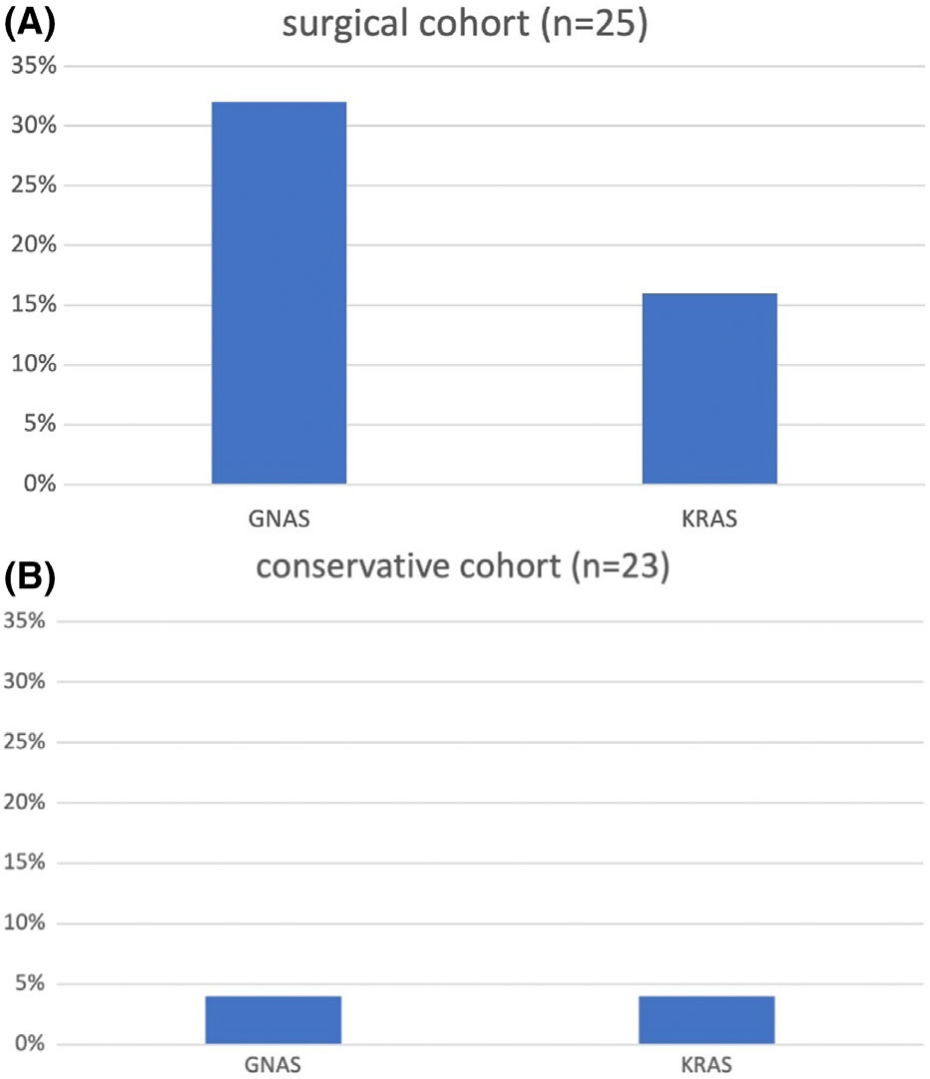
Positivity rate of *KRAS* and *GNAS* mutations in plasma. (A) Positive rates of *KRAS* and *GNAS* mutations in the surgical cohort. (B) Positive rates of *KRAS* and *GNAS* mutations in the conservative cohort.

Among all the patients, 10.4% (*n* = 5) were tested positive for *KRAS* mutations, 18.8% (*n* = 9) for *GNAS* mutations, and 25.0% (*n* = 12) for either *KRAS* and/or *GNAS* mutations in cfDNA (Fig. 1). No tissue samples were analysed.

In the surgical cohort (*n* = 25), 16.0% (*n* = 4) of patients tested positive for *KRAS* mutations, 32.0% (*n* = 8) for *GNAS* mutations, and 40.0% (*n* = 10) for either *KRAS* and/or *GNAS* mutations. In the conservative cohort (*n* = 23), for both *GNAS* and *KRAS* mutations we had each one positive patient (4.3%) (Fig. 1). The *GNAS*‐positive patient's clinical diagnostics in the conservative cohort revealed a new onset of worrisome features during surveillance, necessitating reconsideration of surgery soon after the blood draw. In contrast, the *KRAS‐*positive patient had an additional neurinoma of the appendix.

### 3.2 Correlation with clinicopathological data

Our findings revealed a significant correlation between surgery recommendation and *GNAS* positivity (*P* = 0.024). A significant correlation was found between *KRAS* and worrisome features in branch duct IPMN (*P* = 0.043). In patients without worrisome features, 22 out of 23 patients were *KRAS* negative. The same results were evident for *GNAS*‐negative patients. All 23 patients without worrisome features were *GNAS* negative. The *GNAS* status was additionally associated with the type of IPMN (*P* = 0.031). No further correlations with other clinicopathological parameters were evident (Tables 1 and 2). Notably, the grade of dysplasia did not show a significant correlation with *KRAS* and *GNAS* detection in our sample size of *n* = 25 resected patients.

**Table 1:** *KRAS* mutant status and clinicopathological data. Ca 19‐9, Carbohydrate Antigen 19‐9; CEA, Carcinoembryonic Antigen. Significant *P*‐values < 0.05 are bolded; * for *n* = 8 patients no CA 19‐9 and CEA values were available.

	Patients, *n* = 48	*KRAS* negative, *n* = 43	%	*KRAS* positive, *n* = 5	%	*P*‐value
Age						
≤ 67 years	16	15	93.7	1	6.3	0.652
> 67 years	32	28	87.5	4	12.5	
Gender						
Male	19	16	84.2	3	15.8	0.372
Female	29	27	93.1	2	6.9	
Type of IPMN (patho)						
Main/mixed branch	21	18	85.7	3	14.3	0.641
	27	25	92.6	2	7.4	
Grade of dysplasia (resected IPMN *n* = 25)						
Low grade	23	18	78.3	5	21.7	1.000
High grade	2	2	100.0	0	0.0	
Worrisome features (only branch duct *n* = 31)						
Yes	8	5	62.5	3	37.5	**0.043**
No	23	22	95.7	1	4.3	
Surgery						
Yes	25	21	84.0	4	16.0	0.350
No	23	22	95.7	1	4.3	
CA19‐9, *n* = 40*						
< 37 U·mL^−1^	35	30	85.7	5	14.3	0.355
≥ 37 U·mL^−1^	5	5	100.0	0	0.0	
CEA, *n* = 40*						
< 2 mg·L^−1^	28	25	89.3	3	10.7	0.488
> 2 mg·L^−1^	12	10	83.3	2	16.7	

**Table 2:** *GNAS* mutant status and clinicopathological data. Ca 19‐9, Carbohydrate Antigen 19‐9; CEA, Carcinoembryonic Antigen. Significant *P*‐values < 0.05 are bolded; * for *n* = 8 patients no CA 19‐9 and CEA values were available.

	Patients, *n* = 48	*GNAS* negative, *n* = 39	%	*GNAS* positive, *n* = 9	%	*P*‐value
Age						
≤ 67 years	16	14	87.5	2	12.5	0.697
> 67 years	32	25	78.1	7	21.9	
Gender						
Male	19	13	68.4	6	31.6	0.127
Female	29	26	89.7	3	10.3	
Type of IPMN (patho)						
Main/mixed branch	21	14	66.7	7	33.3	**0.031**
	27	25	92.6	2	7.4	
Grade of dysplasia (resected IPMN *n* = 25)						
Low grade	23	16	69.6	7	30.4	1.000
High grade	2	1	50.0	1	50.0	
Worrisome features (only branch duct *n* = 31)						
Yes	8	6	75.0	2	25.0	0.060
No	23	23	100.0	0	0.0	
Surgery						
Yes	25	17	68.0	8	32.0	**0.024**
No	23	22	95.7	1	4.3	
CA19‐9, *n* = 40*						
< 37 U·mL^−1^	35	28	80.0	7	20.0	0.498
≥ 37 U·mL^−1^	5	5	100.0	0	0.0	
CEA, *n* = 40*						
< 2 mg·L^−1^	28	23	82.1	5	17.9	0.881
> 2 mg·L^−1^	12	10	83.3	2	16.7	

The sensitivity of *GNAS* and *KRAS* mutations for identifying IPMN with a higher risk of malignancy (surgical cohort) is relatively low, at 32.0% and 16.0%, respectively. However, the specificity of a negative result for these mutations in cases recommended for surveillance (surveillance cohort) is notably high, at 95.7% in both instances.

### 3.3 Mutant copies per mL plasma *KRAS* and *GNAS* mutations

**Fig. 2:**
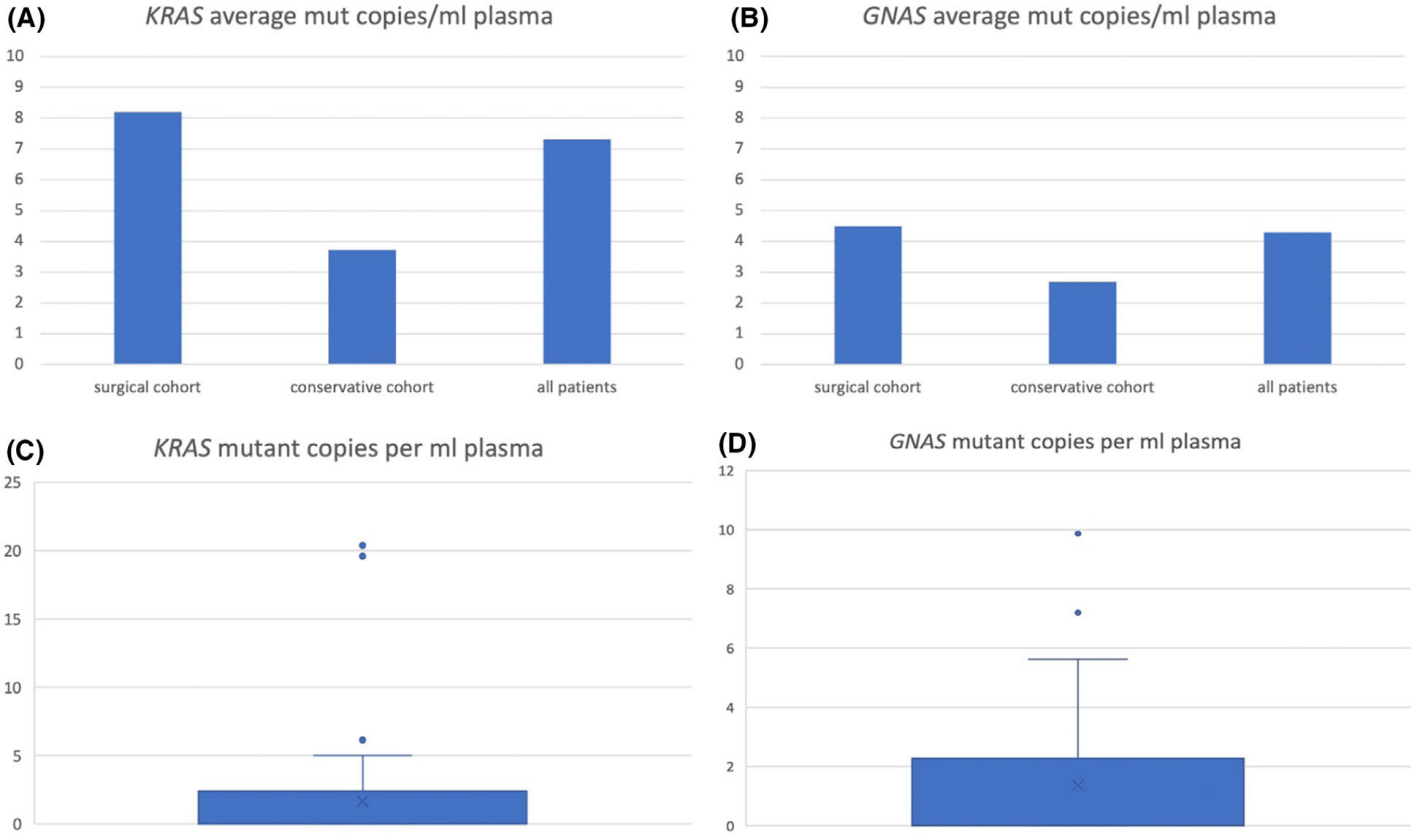
*KRAS* and *GNAS* mutant copies per mL plasma. (A) *KRAS* average mutant copies per mL plasma for *KRAS* positive patients, in the surgical and conservative cohorts and the average of all *KRAS* positive patients. (B) *GNAS* mutant copies per mL plasma for *GNAS* positive patients, in the surgical and conservative cohorts and the average of all *GNAS* positive patients. (C) *KRAS* mutant copies per mL plasma for all patients; the blue box contains the minimum to 75th percentiles, the whisker marks the 95th percentile, and the upper values are the outliers of the dataset; x represents the mean. (D) *GNAS* mutant copies per mL plasma for all patients; the blue box contains the minimum to 75th percentiles, the whisker marks the 95th percentile, and the upper values are the outliers of the dataset; x represents the mean.

For all *GNAS* positive samples, the mean mutant copy number was 4.3 mL^−1^ plasma, and for all *KRAS* positive patients, it was 7.3 mL^−1^ plasma. In the surgical cohort, the mean mutant copy number for *GNAS*‐positive samples was 4.5 mL^−1^ plasma and for *KRAS* 8.2 mL^−1^ plasma, − while in the conservative cohort, the mean mutant copy number for *GNAS* was 2.7 mL^−1^ plasma and for *KRAS* 3.7 mL^−1^ plasma (Fig. 2). The average mutant copies per mL plasma were higher in the surgical cohort throughout both mutations (Fig. 2).

The Mann–Whitney‐*U*‐Test was applied to compute a possible correlation between surgery recommendation and the number of *GNAS* or *KRAS* mutant copies per ml plasma. The correlation between surgery recommendation and *GNAS* mutant copies per mL plasma was significant (*P* = 0.011).

### 3.4 cfDNA concentration in IPMN patients

The mean cfDNA concentration among all patients was 0.5 ng·mL^−1^ plasma. Within the surgical cohort, the mean cfDNA concentration was 0.8 ng·mL^−1^ plasma; in the conservative cohort, it was 0.3 ng·mL^−1^ plasma. We observed a significant correlation (*P* = 0.003) between surgery recommendation and cfDNA concentration. The cfDNA concentration was, on average, 2.2‐fold higher in the surgical cohort.

### 3.5 CECs status

We used the marker‐independent microfluid‐based Parsortix™ cell separation system for CEC detection. No CECs were detected in the analysed cohort of *n* = 31 IPMN patients (*n* = 14 resected and *n* = 17 conservatively managed).

## 4 Discussion

Our study analysed liquid biopsy samples from IPMN patients for *KRAS* and *GNAS* mutations and CECs. Our data show that *GNAS* and *KRAS* mutations (18.8% and 10.4%) can already be found in the cfDNA of IPMN patients in general, concluding that IPMNs, as preneoplastic disease, can shed mutated *KRAS* and *GNAS* cfDNA into the bloodstream.

We could furthermore show that both the *GNAS* positivity rate and mutational load (mutant copies·mL^−1^) were statistically significantly higher in the surgical cohort compared to the surveillance cohort (32% vs. 4% (*P* = 0.024) and 4.5 vs. 2.7 mut copy·mL^−1^ (*P* = 0.011) respectively). For *KRAS*, only the mutation load was higher in the surgical cohort (8.2 vs. 3.7 mut copy·mL^−1^).

In our study, a further interesting observation was the 2.2‐fold increase in total mutant cfDNA concentrations within the surgical cohort (*P* = 0.003), underscoring the potential significance of cfDNA‐based analysis in evaluating malignancy risk. However, it is noteworthy that most resected IPMN (23 out of 25) were pathologically classified as low‐grade dysplasia. In the subset of patients with high‐grade dysplasia (*n* = 2), one exhibited a *GNAS* mutation, while neither showed *KRAS* mutations, limiting the scope for definitive clinical correlations or conclusions. Due to our limited sample size, establishing a significant correlation between *KRAS* or *GNAS* mutations and the grade of dysplasia in resected patients was not feasible. Nonetheless, this preliminary observation suggests that a more extensive study could reveal more definitive patterns. A further limitation of our study is the exclusive analysis of the *GNAS* hotspot mutation R201C, caused by the limited amount of cfDNA found in the IPMN patients. It is important to note that the positivity rate and discrimination power could have potentially been even higher than reported when both *GNAS* R201C and R201H mutations were analysed [20].

Our results align with other mainly smaller liquid biopsy studies on IPMN. Berger et al. [21] had a sample size of *n* = 21 and reported positivity rates for *KRAS* of 0% and *GNAS* of 71% in samples from IPMN patients under surveillance. In contrast, in their resected cohort of previously stored serum samples (*n* = 16), the positive rates were 0% for *KRAS* and 25% for *GNAS*. Hata et al. [22] had a sample size of *n* = 34 and reported positivity rates for *KRAS* of 6% and *GNAS* of 32% in samples from resected IPMN patients. Furthermore, Okada et al. [23] analysed a larger IPMN follow‐up cohort of *n* = 112 patients regarding cfDNA quantification and mutant allele frequency (MAF)—concluding that the MAF of *KRAS* in IPMN patients was significantly increased compared with healthy controls. At the same time, the MAF of *GNAS* did not show significant changes. Finally, Park et al. [24] did detect neither *KRAS* nor *GNAS* mutations in their cohort of *n* = 15 resected IPMN patients, possibly due to the limited amount of input plasma (300–500 μL). None of the studies compared prospectively *KRAS* and *GNAS* mutations in liquid biopsy samples between two IPMN patient cohorts with different clinically IPMN risk stratifications (surgical resected vs. under surveillance) (Table S1).

Reading tissue status, the current literature reports a wide range of positivity rates for *KRAS* and *GNAS* mutations, with *KRAS* mutations detected in 31% to 88% of cases and *GNAS* mutations in 41% to 79% of resected IPMN patients [13, 24, 25, 26, 27, 28, 29]. Similarly, cyst fluid analysis in IPMN patients shows variable positivity rates, ranging from 26% to 82% for *KRAS* and 27% to 66% for *GNAS* mutations [17, 30, 31, 32, 33, 34]. In the pancreatic juice of patients with IPMN, current literature indicates a positive detection rate ranging from 39% to 62% for *KRAS* and 31% to 65% for *GNAS* mutations [29, 35, 36, 37, 38]. Thus, not surprisingly, *KRAS* and *GNAS* mutations in both high‐ and low‐risk IPMN patients are less frequently detected in liquid biopsy than in other analytes. Nevertheless, our study highlights that even in its preneoplastic stage, IPMN can lead to the presence of *KRAS* and *GNAS* mutations in the bloodstream of patients. Blood analytes are more accessible and are also a frequent monitoring tool.

Regarding translational significance, our study indicates that detecting *GNAS* mutations in liquid biopsy may correlate with clinical imaging‐based recommendations for assessing the malignancy risk in IPMN patients. Given the relatively low sensitivity but high specificity of *KRAS* and *GNAS* detection in cfDNA, our primary conclusion is that the absence of these mutations supports a conservative surveillance approach, particularly for branch duct IPMN lacking worrisome features. This suggests that *KRAS* and *GNAS* mutations in liquid biopsy samples from IPMN patients could primarily serve to reinforce the decision for conservative surveillance in cases of branch duct IPMN without worrisome features—which underlines the study's clinical relevance, providing a more transparent basis for risk stratification and management decisions. Within our study cohort, *GNAS* and *KRAS* mutations in cfDNA were each detected in only one case in patients without worrisome features. Notably, the one *GNAS*‐positive patient, initially diagnosed with a branch duct IPMN without worrisome features, had worrisome features identified in subsequent surveillance imaging, leading to a surgical recommendation. Moreover, a shift to *GNAS* positivity could potentially prompt more intensive imaging surveillance. However, this hypothesis warrants further validation in larger study cohorts to solidify its clinical applicability.

In our study, unlike the cfDNA findings, we did not observe any CECs in our IPMN cohort. This contrasts with other studies that reported CEC detection rates in IPMN samples/pancreatic cystic lesions ranging from 33% to 88% [39, 40, 41, 42, 43]. The previous research has utilised various microfluidic‐ and size‐based cell isolation systems to isolate and identify CECs in cystic pancreatic lesions and PDAC, indicating that numerous conditions might be associated with CEC detection [42, 43]. Our study is the first to implement the Parsortix™, a size‐based cell separation system for CEC isolation. This discrepancy suggests that our cell separation method, or the pan‐keratin antibody, might be less effective for isolating and detecting CECs in IPMN cases. Interestingly, our platform has proven capable of detecting CTCs in a significant proportion of PDAC patients [44]. This raises the possibility that CECs and CTCs may differ in their physical properties, potentially affecting their detectability using our current methodology. The lack of standardised CEC and CTC detection methods, especially for malignancies of the pancreas [43], indicates that ctDNA‐based mutation analyses rather qualify for biomarker research in IPMN settings than CEC analysis. Here, sensitive and cost‐effective *KRAS* and *GNAS* based mutation analyses offer a more standardised and objective approach.

## 5 Conclusions

In summary, our study represents the first to investigate the differences in the frequency of *GNAS* and *KRAS* mutations between two distinct IPMN cohorts—one undergoing conservative monitoring and the other with recommendations for surgical intervention. Our findings highlight the potential utility of assessing *KRAS* and *GNAS* mutations in cfDNA to guide IPMN treatment decision‐making. Notably, the absence of these mutations appears to be a reliable indicator for branch duct IPMN patients without worrisome features, supporting a conservative management approach in low‐risk cases. Conversely, a shift to *GNAS* positivity could be considered a significant marker for enhanced imaging surveillance, helping to identify patients at a higher risk of malignant transformation who may require surgical resection. However, it is worth noting that a substantial majority of patients within the surgical cohort exhibited only low‐grade dysplasia, raising questions about the necessity of surgery in these cases. Neither the presence of established worrisome features nor *GNAS* or *KRAS* mutations appear effective in identifying high‐grade dysplasia among IPMN patients, which is the only true indication for surgery. Future research involving larger patient cohorts and longer follow‐up periods is essential to solidify these preliminary observations.

## Conflict of interest

The authors declare no conflict of interest.

## Author contributions

The corresponding author takes full responsibility that all authors on this publication have met the following required criteria of eligibility for authorship: (a) significant contributions to the conception and design, acquisition of data, or analysis and interpretation of data; (b) drafting or revising the article for intellectual content; (c) final approval of the published article; and (d) agreement to be accountable for all aspects of the article thus ensuring that questions related to the accuracy or integrity of any part of the article are appropriately investigated and resolved. Nobody who qualifies for authorship has been omitted from the list. CN, MT, HW and FGU conceived and designed the project. CN, MT, PW, KM, MG, JK, AWB, JRI, FN, TH, KP, HW and FGU acquired the data and provided the samples. CN, MT, HW and FGU analysed and interpreted the data. CN and MT wrote the paper.

## Supporting information


**Table S1.** Comparison of cfDNA analysis data in IPMN.

## Data Availability

The data that support the findings of this study are available from the corresponding author [c.nitschke@uke.de] upon reasonable request.
