# Supplementary material for: KRAS and GNAS mutations in cell‐free DNA and in circulating epithelial cells in patients with intraductal papillary mucinous neoplasms—an observational pilot study
Source: Mol Oncol. 2024 Sep 1;19(7):2144–53. doi: 10.1002/1878-0261.13719 (PMC12234379; doi:10.1002/1878-0261.13719)
Supplement: Supplementary file 1 — Table S1. Comparison of cfDNA analysis data in IPMN. [file MOL2-19-2144-s001.docx]

|  |  |  |  |  |  |  |  | **cfDNA result** | |
| --- | --- | --- | --- | --- | --- | --- | --- | --- | --- |
| **Reference** | **sample size n** | **study design (retrospective/prospective)** | **IPMN (surgical resected/under surveillance)** | **method** | **mutations analyzed** | **plasma/serum volume analysed** | **mean cfDNA** | ***GNAS*** | ***KRAS*** |
| **Berger et al. 2016** | **n=21** | **retrospective** | **surveillance** | **ddPCR** | ***KRAS*: G12D/V; *GNAS* 201C/H** | **2 ml plasma** | **0.3 ng/μl** | **71.4%** | **0.0%** |
|  | **n=16** | **retrospective** | **resected** | **ddPCR** | ***KRAS*: G12D; *GNAS* 201C/H** | **2 ml serum** | **n.f.** | **25.0%** | **0.0%** |
| Okada et al. 2020 | n=112 | prospective | follow up IPMN | pre- amp-ddPCR pools | pool 1: G12D/V/C, G13D; pool2: G12R/S/A, G13C; *GNAS* R201C/H | 3-4 ml plasma | 14.4 ng/ml | mutation status n.d.; MAF not different from cntrl | mutation status n.d.; MAF sign increased in IPMN comp. to cntrl |
| **Hata et al. 2020** | **n=34** | **prospective** | **resected** | **ddPCR** | ***KRAS*: G12A/C/D/R/S/V, G13D; *GNAS*: R201C/H** | **4 ml plasma** | **median 7.5 ng/ml** | **32.0%** | **6.0%** |
| Park et al. 2023 | n=15 | retrospective | resected | NGS | QIAseq, custom panel 22 genes | 300-500 μl plasma | 136.6 pg/μl | 0.0% | 0.0% |
| Levink et al. 2023 | n=1 | prospective | resected | NGS | panel* | 1.9 ml plasma | 87.3 ng/μl | n.a. | 100.0% |
| **Nitschke et al.** | **n=48** | **prospective** | **surveillance** | **ddPCR** | ***KRAS*: G12A/C/D/R/S/V, G13D; *GNAS*: R201C** | **3-6 ml plasma** | **0.3 ng/ml** | **4.3%** | **4.3%** |
|  | **n=48** | **prospective** | **resected** | **ddPCR** | ***KRAS*: G12A/C/D/R/S/V, G13D; *GNAS*: R201C** | **3-6 ml plasma** | **0.8 ng/ml** | **32.0%** | **16.0%** |

**Supplementary Table 1: Comparison of cfDNA analysis data in IPMN**

NGS: next-generation sequencing

n.a.: not analyzed

cntrl: control sample, MAF: mutant allele frequency

n.f.: not found

n.d.: not detected

* different panels compared
